# Atypical Phenotype of Predominant Autoimmune Cytopenia and Impaired Perforin Expression in XMEN Syndrome

**DOI:** 10.1155/jimr/3161910

**Published:** 2025-10-24

**Authors:** Hana Grombirikova, Adam Markocsy, Anna Kocurkova, Jan Blatny, Marcela Vlkova, Peter Slanina, Eva Hlavackova, Veronika Fiamoli, Helena Schneiderova, Adam Klocperk, Dita Ricna, Eva Fronkova, Jan Kral, Anna Salingova, Milos Jesenak, Tomas Freiberger

**Affiliations:** ^1^ Centre for Cardiovascular Surgery and Transplantation, Brno, Czech Republic, cktch.cz; ^2^ Faculty of Medicine, Masaryk University, Brno, Czech Republic, muni.cz; ^3^ Centre for Primary Immunodeficiencies, Department of Paediatrics and Adolescent Medicine, Jessenius Faculty of Medicine, Comenius University in Bratislava, University Hospital in Martin, Martin, Slovakia, uniba.sk; ^4^ Institute of Clinical Immunology and Medical Genetics, Jessenius Faculty of Medicine, Comenius University in Bratislava, University Hospital in Martin, Martin, Slovakia, uniba.sk; ^5^ Department of Biophysics of Immune System, Institute of Biophysics of the Czech Academy of Sciences, Brno, Czech Republic, ibp.cz; ^6^ Department of Paediatric Haematology and Biochemistry of University Hospital Brno and Faculty of Medicine, Masaryk University, Brno, Czech Republic, muni.cz; ^7^ Hospital Bory, Bratislava, Slovakia; ^8^ Institute of Clinical Immunology and Allergology, St. Anne’s University Hospital in Brno, Brno, Czech Republic, fnusa.cz; ^9^ Department of Paediatrics, University Hospital Brno, Brno, Czech Republic, fnbrno.cz; ^10^ Department of Immunology, 2nd Faculty of Medicine, Charles University and University Hospital in Motol, Prague, Czech Republic, cuni.cz; ^11^ CLIP Laboratory Centre, Department of Paediatric Haematology and Oncology, Second Faculty of Medicine, Charles University and University Hospital Motol, Prague, Czech Republic, cuni.cz; ^12^ Centre for Medical Genetics and Reproductive Medicine, GENNET, Prague, Czech Republic; ^13^ Department of Laboratory Medicine, Centre of Inherited Metabolic Diseases, National Institute of Children’s Diseases, Bratislava, Slovakia; ^14^ Centre for Primary Immunodeficiencies, Department of Pulmonology and Phthisiology, Jessenius Faculty of Medicine, Comenius University in Bratislava, University Hospital in Martin, Martin, Slovakia, uniba.sk

**Keywords:** autoimmune haemolytic anaemia, autoimmune cytopenia, congenital disorders of glycosylation, immune thrombocytopenia, *MAGT1*, perforin, XMEN

## Abstract

X‐linked immunodeficiency with magnesium defect, Epstein–Barr virus (EBV) infection, and neoplasia (XMEN) is caused by a pathogenic variant in the magnesium transporter 1 (*MAGT1*) gene. The defect leads to impaired N‐glycosylation which affects various immune processes. In this study, we described the disease course, clinical features and laboratory parameters observed in six patients from three families diagnosed with XMEN syndrome. They exhibit heterogeneous clinical manifestation while displaying typical laboratory signs of the disease, including decreased surface expression of NKG2D and CD28 on CD8^+^ T‐cells and NK cells, as well as defects in the N‐glycosylation of transferrin. We identified two novel variants in the cohort: a frameshift variant c.444dup in exon 3, and a splicing variant c.998‐20_1008del. Notably, a patient with the c.444dup variant presented with severe autoimmune cytopenia as an isolated manifestation of the disease, while his younger brother, carrying the same variant, exhibited predominantly mild skin infections. These findings illustrate varying degrees of severity in penetrance and highlight that some patients may exhibit only partial symptoms. Furthermore, our study confirmed defects in perforin expression in XMEN syndrome. We observed a significant reduction in perforin expression within CD8^+^ T‐cells and NK cells which may lead to increased susceptibility to recurrent infections and autoimmune complications frequently observed in XMEN patients.

## 1. Introduction

The magnesium transporter 1 (MAGT1), initially described as an Mg^2+^ specific ion transporter, is a protein that plays a key role as a regulator of Mg^2+^ as a second messenger during T‐cell receptor activation [1, 2]. MAGT1 and its protein homologue TUSC3 are both non‐catalytic subunits of the oligosaccharyltransferase (OST) complex, facilitating STT3B‐dependent glycosylation. MAGT1 promotes the N‐linked glycosylation of specific glycoproteins crucial for immune signalling and function [3–5].

Hemizygous loss‐of‐function variants in the *MAGT1* gene are associated with X‐linked immunodeficiency with magnesium defect, Epstein–Barr virus (EBV) infection, and neoplasia (XMEN) disease [6, 7]. MAGT1 deficiency leads to defective activation of CD8^+^ T‐cells and NK‐cells due to decreased Mg^2+^ influx as well as impaired glycosylation and decreased surface expression of the activator receptors natural killer group 2, member D (NKG2D), CD28 and CD70 [8, 9].

XMEN disease is a primary immunodeficiency that falls under the category of immune dysregulation disorders [10]. Clinical manifestations and laboratory findings are highly heterogeneous, with no established genotype‐phenotype correlations [11]. XMEN patients often suffer from recurrent ear and sinopulmonary infections, and recurrent viral infections (herpes simplex and molluscum contagiosum). Chronic lymphadenopathy, splenomegaly and a heightened risk of EBV‐associated B‐cell malignancies are typical hallmarks of the disease. In some patients, the clinical presentation of XMEN syndrome may mimic autoimmune lymphoproliferative syndrome [12]. From a laboratory standpoint, patients often exhibit cytopenias, transient elevations in creatine kinase (CK), aspartate aminotransferase (AST) and/or alanine aminotransferase (ALT). The immune profile of the disease includes hypogammaglobulinemia (IgG and IgA), increased number of total B‐cells, CD4^+^ T‐cell lymphopenia, an inverted CD4/CD8 ratio, increased percentage of CD4^–^CD8^–^TCRαβ^+^ T‐cells (αβDNTs) and decreased expression of NKG2D receptor [5]. Interestingly, defects in perforin expression and cytotoxicity reduction in CD8^+^ T‐cells and NK cells were observed by Wang et al. [13] in 2024.

## 2. Methods

### 2.1. Genetic Testing

Genomic DNA samples from probands in all families were analysed using next generation sequencing (NGS). Patients P1, P3 and P4 were analysed using NGS targeted to genes associated with inborn errors of immunity (IEI), based on the recommendations of the International Union of Immunological Societies [14]. Patient P6 was analysed using whole exome sequencing (WES). The comprehensive list of targeted genes, details of the library preparation and sequencing specifics are available in the Supporting information. IEI targeted sequencing was performed on the NextSeq 500 platform (Illumina, San Diego, CA) whereas WES was conducted on the NovaSeq X Plus platform (Illumina, San Diego, CA). To validate the presence of the identified variants in the probands and to screen for these variants in their symptomatic and asymptomatic relatives (also in patients P2 and P5), Sanger sequencing was subsequently employed (primers available on request). Detection of variants in family members enabled the evaluation of inheritance patterns and the assessment of risks for individuals carrying hemizygous or heterozygous variants.

### 2.2. Immunophenotyping

To perform surface staining, 100 µL of anticoagulated heparinised blood was incubated with the appropriate mixture of antibodies. The expression of NKG2D and CD1d cell receptors was assessed using anti‐CD56 PE (clone N901, Beckman Coulter, Inc.), CD16 PE (Clone 3G8, Beckman Coulter, Inc.), CD3 FITC (clone UCHT1, Beckman Coulter, Inc.), NKG2D APC (Clone 149810, R&D Systems), CD8 Alexa Fluor 700 (Clone MEM‐31, Exbio), CD19 PE‐Cy7 (clone HIB19, Sony), CD45 PE‐DL 594 (clone MEM‐28, Exbio) and CD1d BV510 (Clone CD1d42, BD Horizon). Additionally, CD28 staining was conducted using the DURAClone IM T‐Cell Subsets Tube from Beckman Coulter, while B‐cell subsets were determined using the DURAClone IM B‐Cells from Beckman Coulter. Surface expression of CD28 and NKG2D was measured using the Navios EX Flow Cytometer (Beckman Coulter) in patients P1–P5, and the LSRFortessa Flow Cytometer (Becton Dickinson) in patient P6.

### 2.3. Analysis of Perforin Expression and N‐Glycosylation in CD8^+^ and NK Cells

To detect changes in perforin and N‐glycosylation, blood samples from patients and healthy volunteers were collected into heparin. The PBMC fractions were separated using Ficoll‐Paque (Cytiva, cat. no. 17544203) density gradient centrifugation. PBMCs were diluted to a concentration 3 × 10^6^ cells/mL, and 100 µL of the cell suspension was pipetted per sample. Initially, the viability of the cells was assessed by staining with the LIVE/DEAD Fixable Aqua DeadCell Stain Kit (Invitrogen, cat. no. L34957A) for 30 min at 4°C. Subsequently, the PBMCs were washed with PBS and extracellular markers were labelled for 30 min at 4°C by specific conjugated antibodies: SingleFlowEx CD45 PE‐Cy7 (Exbio, cat. no. ED7207), Anti‐Hu CD3 PE‐DyLight 594 (Exbio, cat. no. T5‐202‐T100), Anti‐Hu CD14 PerCP‐Cy5.5 (Exbio, cat. no. T9‐293‐T100), Anti‐Hu CD16 APC‐Cy7 (Exbio, cat. no. T4‐646‐T100) and BD Horizon APC‐R700 Mouse Anti‐Human CD8 (BD Biosciences, cat. no. 565165). Following incubation, the PBMCs were fixed and permeabilized with eBioscience Foxp3/Transcription Factor Staining Buffer Set (ThermoFisher Scientific, cat. no. 00‐5523‐00) according to the manufacturer’s recommendation. Briefly, the Fixation/Permeabilization Concentrate was diluted in eBiosciences Fixation/Perm Diluent (1:3), and 200 µL of the diluted fixation solution was added to the PBMCs for 30 min at RT. After fixation, cells were washed with permeabilization buffer pre‐diluted in distilled water (1:9), and intracellularly stained by BD Horizon BV421 Mouse Anti‐Human Perforin (BD Biosciences, cat. no. 563393) and fluorescently labelled lectins diluted in Permeabilization buffer (30 min at 4°C). Specifically, *Phaseolus Vulgaris* leucoagglutinin (PHA‐L), Fluorescein (Vector Laboratories, cat. no. FL‐1111‐2, dilution 1:100) and *Sambucus Nigra* lectin (SNA, EBL), CY5 (Vector Laboratories, CL‐1305‐1, dilution 1:100) were used. PHA‐L recognises complex‐type N‐glycans containing the specific sequence: Galβ1‐4GlcNAcβ1‐2(Galβ1‐4GlcNAcβ1‐6)Manα1‐R, also known as 2,6 branch. SNA is employed for the detection of α2‐6‐sialylated products. Samples were measured using the Navios EX Flow Cytometer (Beckman Coulter), and the data were analysed in Kaluza Analysis Software (Beckman Coulter). CD8^+^ cells were gated as LIVE/DEAD^low^, CD45^+^, CD14^−^, CD16^−^, CD3^+^ and CD8^+^, while NK cells were gated as LIVE/DEAD^low^, CD45^+^, CD14^−^, CD16^+^ and CD3^−^. The median fluorescence intensity (MFI) for PHA‐L and SNA or perforin in these two populations was detected. Patient samples were measured in technical duplicates and results are shown in bar graphs or scatter dot plots as mean with SEM in comparison to 3–6 repetitions of healthy control individuals.

### 2.4. Transferrin Isoelectric Focusing (IEF) Analysis

For transferrin analysis, 10 µL of serum was incubated for 30 min with an Fe(III)‐citrate and NaHCO_3_ solution to saturate transferrin with Fe(III). Subsequently, 1 µL of the resulting supernatant was applied to a polyacrylamide gel with a pH gradient of 5–8 (PhastGel, GE Healthcare Bio‐Sciences) and analysed on a PhastSystem (GE Healthcare). Transferrin isoforms were detected by immunofixation using the Polyclonal Rabbit Anti‐Human Transferrin antibody (DAKO, Denmark) for 60 min, followed by overnight saline washing and PhastGel Blue R staining.

## 3. Results

### 3.1. Patients

We studied the disease course, clinical and laboratory parameters of six patients diagnosed with XMEN syndrome by molecular‐genetic testing. The patients´ clinical manifestations are summarised in Table 1 and selected laboratory parameters are shown in Table 2. Below is a detailed description of the disease development in patients.

**Table 1:** Clinical manifestation of XMEN patients.

Patient	Patient 1	Patient 2	Patient 3	Patient 4	Patient 5	Patient 6
Variant (NM_032121.5)	c.444dupT	c.444dupT	c.409C>T	c.409C>T	c.409C>T	c.998‐20_1008del
Infectious complications						
Recurrent sinusitis	Y	N	Y	Y	N	N
Recurrent otitis media	Y	N	N	N	N	Y
Recurrent bronchitis	Y	N	Y	N	Y	Y
Severe pneumonia	N	N	N	Y	Y	Y
Myositis/muscle abscess	N	N	N	Y	N	Y
Severe gastrointestinal	N	N	N	Y	Y	Y
Meningitis	N	N	N	Y	N	N
Skin infection						
Warts	N	N	N	N	N	Y
Molluscum contagiosum	N	Y	N	N	N	N
Pityriasis versicolour	N	Y	N	N	N	N
Recurrent HSV infection	N	N	Y	Y	Y	N
Autoimmune disease						
Severe cytopenia	Y	N	N	N	N	N
Anaemia	Y	N	Y	N	Y	N
Thrombocytopenia	Y	N	Y	N	Y	N
Neutropenia	Y	N	N	N	Y	Y
Clinical findings						
Hepatomegaly	N	N	Y	N	N	N
Splenomegaly	N	N	N	Y	Y	N
Lymphadenopathy	N	N	Y	Y	Y	N
EBV status						
Status post EBV infection	Y	Y	Y	Y	Y	Y
Malignancy						
EBV associated lymphoma	N	N	N	Y	N	N

*Note:* The table lists symptoms observed in patients at any point during the disease. Severe cytopenia is defined by the presence of at least one of the following conditions: severe anaemia (haemoglobin <80 g/L), severe thrombocytopenia (platelet count <50 × 10^9^/L), or severe neutropenia (neutrophil count <0.5 × 10^9^/L). EBV status was monitored through blood serology (EBV‐VCA IgM, EBV‐VCA IgG, EBV‐EBNA‐1 IgG, anti‐EBV EBNA IgM, anti‐EBV‐EA IgM and anti‐EBV‐EA IgG) and PCR testing of oral swab (in P1 and P2), blood (lymphocytes) and urine samples (in P3–P5). N, no; Y, yes.

Abbreviations: EBV, Epstein–Barr virus; HSV, herpes simplex virus.

**Table 2:** Laboratory parameters of XMEN patients.

Laboratory findings	Patient 1	Patient 2	Patient 3	Patient 4	Patient 5	Patient 6
IgG (g/L)	3.44 (7.50–15.60)	4.87 (7.50–15.60)	4.70 (7.50–15.60)	4.85 (7.50–15.60)	7.11 (7.50–15.60)	9.00 (7.70–13.60)
IgA (g/L)	0.35 (0.82–4.53)	0.33 (0.82–4.53)	0.29 (0.82–4.53)	0.64 (0.82–4.53)	0.46 (0.82–4.53)	0.40 (0.90–2.90)
ALT (µkat/L)	0.66 (<0.58)	1.22 (<0.58)	4.70 (<0.85)	1.70 (<0.85)	5.10 (<0.85)	1.80 (0.17–0.78)
AST (µkat/L)	0.49 (<0.60)	0.81 (<0.60)	2.80 (<0.85)	0.70 (<0.85)	1.40 (<0.85)	0.50 (0.16–0.72)
GGT (µkat/L)	0.46 (0.10–0.70)	0.33 (0.10–0.70)	0.20 (<0.92)	1.50 (<0.92)	0.80 (<0.92)	0.40 (0.14–0.84)
Creatine kinase (µkat/L)	2.27 (<2.85)	NA	6.20 (<2.85)	3.30 (<2.85)	1.20 (<2.85)	4.00 (0.41–3.24)
T‐cells and NK cells						
CD3 T‐cells (%)	44.80 (55.00–83.00)	42.80 (55.00–83.00)	45.00 (55.00–83.00)	38.00 (55.00–83.00)	45.00 (52.00–78.00)	47.00 (52.00–90.00)
CD4 T‐cells (%)	18.90 (28.00–57.00)	16.30 (28.00–57.00)	26.00 (28.00–57.00)	21.00 (28.00–57.00)	19.00 (25.00–48.00)	22.00 (20.00–65.00)
CD4/CD8 ratio	0.97 (0.7–3.0)	0.74 (0.70–3.0)	2.00 (0.70–3.00)	1.90 (0.70–3.00)	1.00 (0.70–3.00)	1.20 (1.00–3.00)
CD28 surface expression on CD8^+^ T‐cells subpopulations (MFI)	3.00 (>4.78)	3.30 (>4.78)	4.07 (>4.78)	2.80 (>4.78)	3.14 (>4.78)	1108.00 (>1233.00)
DNT‐cells (%)	1.96 (<2.50)	1.26 (<2.50)	4.30 (<2.50)	5.20 (<2.50)	4.00 (<2.50)	6.00 (<5.00)
NKG2D surface expression on CD8^+^ T‐cells (MFI)	0.30 (>0.58)	0.31 (>0.58)	0.15 (>0.58)	0.17 (>0.58)	0.17 (>0.58)	27.00 (>416.00)
NKG2D surface expression on NK cells (MFI)	0.18 (>0.43)	0.18 (>0.43)	0.16 (>0.43)	0.16 (>0.43)	0.15 (>0.43)	0.00 (>240.00)
CD1d surface expression on CD8^+^ T‐cells (MFI)	0.45 (>0.33)	0.47 (>0.33)	0.30 (>0.33)	0.35 (>0.33)	0.32 (>0.33)	NA
CD1d surface expression on NK cells (MFI)	0.43 (>0.28)	0.46 (>0.28)	0.26 (>0.28)	0.36 (>0.28)	0.29 (>0.28)	NA
NK cells (%)	18.90 (7.00–31.00)	7.90 (7.00–31.00)	3.00 (7.00–31.00)	11.00 (7.00–31.00)	6.00 (6.00–27.00)	3.50 (6.00–28.00)
B‐cells						
B‐cells (%)	33.70 (6.00–19.00)	43.90 (6.00–19.00)	50.00 (6.00–19.00)	49.00 (6.00–19.00)	48.00 (8.00–24.00)	45.00 (9.40–22.80)
Naive B‐cells (%)	95.50 (75.20–86.70)	96.80 (75.20–86.70)	95.60 (65.60–79.60)	94.20 (58.00–72.10)	94.10 (75.20–86.70)	93.90 (51.30–82.50)
Marginal zone B‐cells (%)	1.60 (4.60–10.20)	1.80 (4.60–10.20)	2.20 (7.40–13.90)	2.60 (13.40–21.40)	2.70 (4.60–10.20)	3.20 (4.60–18.20)
Transitional B‐cells (%)	7.50 (3.90–7.80)	7.00 (3.90–7.80)	13.30 (3.00–5.90)	11.10 (1.00–3.60)	22.50 (3.90–7.80)	6.40 (1.40–13.00)
Memory B‐cells (%)	0.80 (3.30–9.60)	0.60 (3.30–9.60)	1.20 (7.20–12.70)	2.20 (9.20–18.90)	1.90 (3.30–9.60)	1.30 (8.70–25.60)
Plasmablasts (%)	0.00 (0.30–1.70)	0.00 (0.30–1.70)	0.10 (0.60–1.60)	0.00 (0.60–1.60)	0.10 (0.30–1.70)	0.00 (0.60–6.50)

*Note:* The patients’ values with reference range specific for their age in brackets are displayed. Surface expression of CD28 and NKG2D was measured using the Navios EX Flow Cytometer (Beckman Coulter) in patients P1–P5, and the LSRFortessa Flow Cytometer (Becton Dickinson) in patient P6. Therefore, the values and reference ranges differ between P1–P5 and P6. Relative values of CD3^+^ and CD4^+^ were reduced in all monitored patients. Patients P1 and P2 exhibited an inverted CD4/CD8 T‐cell ratio. Decreased CD28 expression was observed in populations of naïve memory and effector CD8^+^ T‐cells in all patients. Enhancement of DNT was detected in P3–P6. NKG2D expression was decreased on CD8 T‐cells and NK cells. CD1d expression on CD8^+^ and NK cells in patients P3–P5 was near the lower limit of the normal reference range. Slight deviations below this threshold were noted in CD1d expression on CD8^+^ cells (P3 and P5) and NK cells (P5). All patients had an increased number of B‐cells, naïve B‐cells, and conversely, a decreased number of memory B‐cells, plasmablasts and marginal zone B‐cells.

Abbreviations: ALT, alanine aminotransferase; AST, aspartate aminotransferase; DNT, double‐negative (CD3^+^CD4^−^CD8^−^TCRα/β) T‐cells; GGT, gamma‐glutamyl transferase; MFI, median fluorescence intensity; NA, not available.

Patient P1, a 20‐year‐old man, exhibited a complex medical history, beginning with an episode of severe autoimmune haemolytic anaemia (AIHA) at the age of 4 years, followed by numerous treatment‐resistant attacks of immune thrombocytopenia (ITP) and AIHA during the disease course. At 12 years, the patient faced severe leukopoenia and neutropenia during a pertussis infection, with spontaneous resolution. AIHA and ITP attacks required extensive treatment. He received multiple transfusions and underwent treatment with corticosteroids, intravenous immunoglobulins (IVIG), rituximab and eltrombopag olamine (thrombopoietin receptor agonist), however with poor effect, which necessitated a splenectomy. Due to haemorrhagic complications of ITP at the age of 15 years presented with bleeding into cavernomas together with haematuria, romiplostim (fusion thrombopoietin peptide analogue) was administered. Despite these combined interventions, recurrent ITP attacks persisted. Due to the disease course described above, genetic testing was conducted, revealing a diagnosis of XMEN syndrome. At 16 years of age, the treatment with mycophenolate mofetil significantly modified cytopenic symptoms. Consequently, only regular IVIG administration has been used to treat persistent hypogammaglobulinemia.

Patient P2, a 17‐year‐old man and brother of patient P1, was diagnosed with XMEN syndrome based on segregation analysis of the variant found in his brother. He has been experiencing only mild symptoms of the disease presented with molluscum contagiosum and pityriasis versicolour on his lower extremities and trunk, respectively. The patient is currently not undergoing any treatment.

Patient P3, a 22‐year‐old man, was referred to an immunologist from a young age for recurrent obstructive bronchitis, bronchial asthma and recurrent herpetic infections (mostly herpes labialis). He had frequent episodes of abdominal pain and persistent cervical lymphadenopathy. He was treated with facilitated subcutaneous immunoglobulin replacement therapy (fSCIg) and antiviral drugs for exacerbations of herpetic infections. Due to the phenotype of common variable immunodeficiency and positive family history, genetic testing was performed, revealing a diagnosis of XMEN syndrome.

Patient P4, a 39‐year‐old man and the brother of patient P3, had recurrent herpetic infections and a history of seizures during childhood. At the age of 29 years, he overcame terminal ileitis with the development of abdominal lymphadenopathy and at 32 years he underwent surgery due to left frontal sinus osteoma. After surgery, he developed an epidural brain abscess with post‐puncture complication of purulent pneumococcal meningitis. Afterwards, splenomegaly and cervical lymphadenopathy with histological signs of atypical benign lymphoproliferation occurred. Chest CT scan showed a single nodularity in left lung. He was diagnosed with XMEN syndrome based on segregation analysis of the pathogenic variant in *MAGT1* gene found in his brother. After the diagnosis, chronic EBV and CMV viremia in blood and urine were excluded. He was treated with fSCIg. At the age of 38 years, he developed proximal paresis of the right lower limb due to chronic myositis confirmed by a biopsy of musculus iliopsoas. EBV‐associated oncological complication manifested at the same age represented by classical Hodgkin lymphoma, nodular sclerosis type.

Patient P5, a 15‐year‐old man and maternal cousin of patients P3 and P4, was referred to an immunologist due to atopic dermatitis, allergic rhinitis and bronchial asthma. He underwent recurrent respiratory tract infections and herpetic infections. Abdominal laparoscopic surgery was performed due to gangrenous appendicitis with peritonitis. At that time, he was followed up by an endocrinologist due to short stature with lower values of cortisol and insulin‐like growth factor 1 (IGF‐1) and by a gastroenterologist due to asthenia and idiopathic hepatopathy (repeatedly negative testing for all the hepatotropic viral and bacterial pathogens and negativity of autoantibodies associated with autoimmune hepatitis). He was diagnosed with XMEN syndrome based on segregation analysis of the familial pathogenic variant in *MAGT1* gene. Currently, the liver biopsy is planned.

Patient P6, a 15‐year‐old male, has experienced recurrent lower respiratory tract infections, including cases of pneumonia necessitating antibiotic treatment, a fulminant course of viral gastroenteritis, and recurrent otitis media infections since childhood. At the age of 8, he developed a large femoral abscess with sepsis caused by *Streptococcus* sp., requiring surgical intervention and antibiotic treatment. From this age, he also began suffering from severe and treatment‐recalcitrant skin warts, primarily affecting his fingers. At 11 years, he contracted an EBV infection presenting with an infectious mononucleosis phenotype. The patient’s severe immunodeficiency phenotype prompted clinical exome analysis, resulting in the identification of XMEN syndrome. Following his diagnosis, initiation of subcutaneous immunoglobulin (SCIG) therapy has been started to reduce the infectious burden.

### 3.2. Immunophenotyping

Patients’ samples were analysed repeatedly throughout the course of the disease, and the test results are presented in Table 2. Whenever possible, we report results from tests conducted before any treatment was administered to the patients. However, patient P1 was not suspected of having any immune deficiency for an extended period, resulting in some values not being obtained before treatment initiation. CD3^+^ and CD4^+^ lymphopenia was observed in most examined patients. Patients P1 and P2 also exhibited an inverted CD4/CD8 T‐cell ratio. Additionally, an increased number of B‐cells, and naïve B‐cells were observed, accompanied by a reduction in memory B‐cells and marginal zone B‐cells across all patients. Decreased levels of IgG and IgA were observed in most monitored patients. After confirmation of a diagnosis of XMEN syndrome in patients, typical features of the disease were analysed. Diminished CD28 expression was observed in both naïve memory and effector CD8^+^ T‐cell populations among all tested patients. Enhancement of double‐negative (CD3^+^CD4^−^CD8^−^TCRα/β) T‐cells (DNT) was discerned in four patients (P3–P6). Reduced NKG2D expression on CD8^+^ and NK cells was found in all patients. CD1d expression on CD8^+^ and NK cells in patients P3–P5 was near the lower limit of the normal reference range. Slight deviations below this threshold were noted in CD1d expression on CD8^+^ cells (P3 and P5) and NK cells (P5).

### 3.3. Genetic Testing

Given the 11‐year history of persistent autoimmune cytopenias in patient P1, dating back to his early childhood, an IEI was suspected. Therefore, targeted NGS was performed on IEI‐associated genes. The analysis revealed a novel hemizygous frameshift variant, NM_032121.5:c.444dup;p.(Ala149Cysfs ^∗^6), located within exon 3 of the *MAGT1* gene. Confirmation of the variant’s presence, along with subsequent analysis of DNA samples from the patient’s parents and siblings, was performed using Sanger sequencing. The variant was identified in the patient’s mother and younger brother P2, who was previously considered healthy (Figure 1a).

Figure 1Disease‐causing variants in *MAGT1* found in three families of patients with XMEN disease. The variants are located in exon 3, constituting the thioredoxin domain of *MAGT1*, and in exon 9, constituting the oligosaccharyl transferase complex, subunit OST3/OST6 domain of *MAGT1*. (a) A novel variant c.444dupT was identified in two brothers (P1 and P2) in a hemizygous state, and their mother in a heterozygous state. While the older brother suffered from episodes of severe AIHA and ITP, the only symptom displayed by the younger brother was a molluscum contagiosum rash. (b) A hemizygous variant c.409C >T was identified in two brothers (P3 and P4) and their maternal cousin P5 suffering from various symptoms of XMEN syndrome. This variant was also found in their mothers (II.2 and II.3) in a heterozygous state. Their brother (II.5) reportedly succumbed to stomach cancer at the age of 50, however, the cause and age of death of their second brother remain unknown. Unfortunately, part of the family has not cooperated well and have been unwilling to undergo any examination. NT, not tested. (c) A novel variant c.998‐20_1008del was identified in a male patient in a hemizygous state. This variant was also found in his mother and sister (I.2 and II.3) in a heterozygous state.

**Figure:**
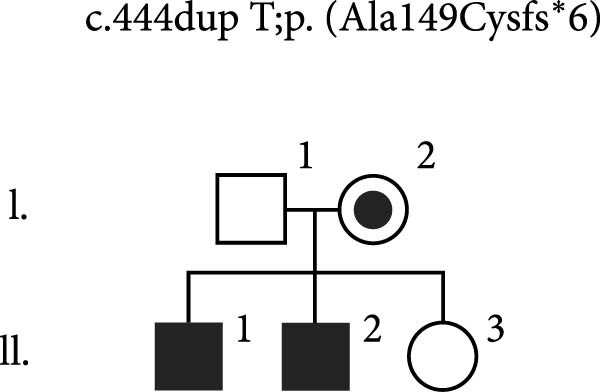
(a)

**Figure:**
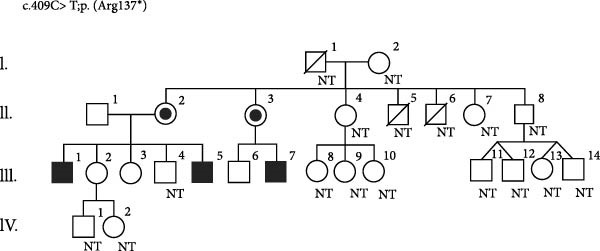
(b)

**Figure:**
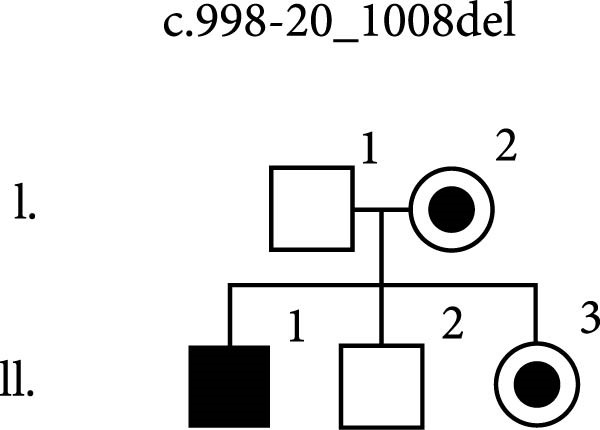
(c)

The severity of the disease symptoms, along with its apparent hereditary nature evidenced by three family members exhibiting similar symptoms, prompted similar genetic analysis within the family of patients P3, P4 and P5. Specifically, the DNA sample from patient P3 underwent targeted NGS focusing on IEI‐associated genes. The analysis revealed a nonsense hemizygous variant, NM_032121.5:c.409C >T;p.(Arg137 ^∗^), which was later confirmed by Sanger sequencing in the patient. This variant was also detected by Sanger sequencing in his similarly affected brother (P4) and maternal cousin (P5) in hemizygous form, as well as in their mothers in heterozygous form. While several other blood relatives tested negative for the presence of the variant by Sanger sequencing, others have refused to undergo the examination (Figure 1b).

Patient P6’s DNA sample was analysed using clinical exome NGS, revealing a novel hemizygous variant, NM_032121.5:c.998‐20_1008del, spanning the intron 8–exon 9 boundary of *MAGT1*, disrupting the acceptor splice site, and thus causing aberrant splicing. Although the predicted effect on the protein appears to be a frameshift, the variant primarily disrupts canonical acceptor splice site positions, which is a well‐established pathogenic mechanism. The variant was identified in the patient’s mother and his sister (Figure 1c).

### 3.4. Perforin Expression Analysis

In individuals with XMEN syndrome, defects in perforin expression and a decrease in cytotoxic activity within CD8^+^ T‐cells and NK cells has been reported [9, 13]. Accordingly, we investigated the expression of perforin within these subpopulations. Cytotoxic T lymphocytes and NK cells from healthy donors showed variable perforin expression and could be subdivided into perforin^low^ and perforin^high^ subpopulations (Figures 2a–h and 3a–h) [15, 16].

Figure 2Detection of perforin expression in CD8^+^ lymphocytes. Perforin expression was detected by flow cytometry after fixation and permeabilization. CD8^+^ cells were gated as LIVE/DEAD^low^, CD45^+^, CD14^−^, CD16^−^, CD3^+^ and CD8^+^. Representative histogram of healthy donor without stimulation (a), representative histogram of patient without stimulation (b), representative histogram of healthy control after stimulation (c) and representative histogram of patient after stimulation (d). The expression of perforin is variable, thus the histograms were divided into perforin^low^ and perforin^high^ sections and evaluated separately. Results are shown in the graphs as MFI of perforin^low^ (e), MFI of perforin^high^ (f), % of perforin^low^ (g) and % of perforin^high^ (h). Results are presented as dot plots connected with lines to compare state before and after stimulation. Statistical significance was evaluated using the Mann–Whitney test in GraphPad Prism software; *n* = 5 (patients P1–P5; data from P6 were obtained later and are not included in this graph but showed similarly reduced values);  ^∗^ *p* < 0.05. MFI, median fluorescence intensity.

**Figure:**
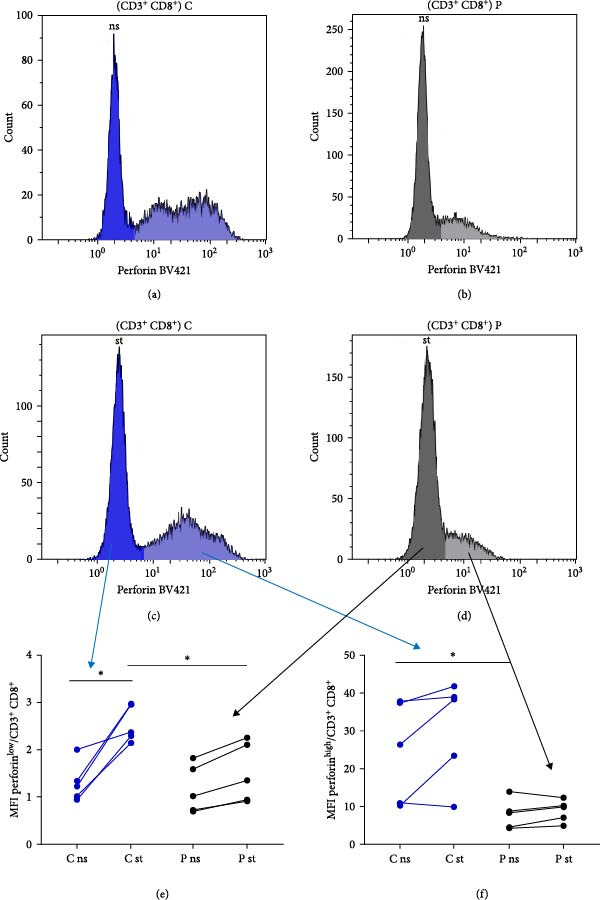

**Figure:**
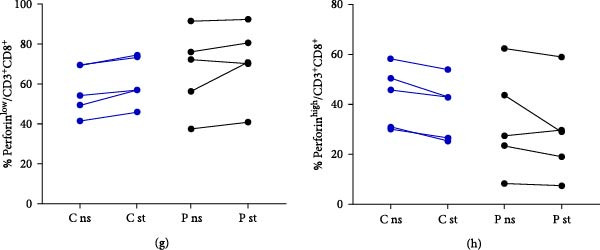

Figure 3Detection of perforin expression in NK cells. Perforin expression was detected by flow cytometry in NK cells after fixation and permeabilization. NK cells were gated as LIVE/DEAD^low^, CD45^+^, CD14^−^, CD16^+^ and CD3^−^. Representative histogram of healthy donor without stimulation (a), representative histogram of patient without stimulation (b), representative histogram of healthy control after stimulation (c) and representative histogram of patient after stimulation (d). The expression of perforin is variable, thus the histograms were divided into perforin^low^ and perforin^high^ sections and evaluated separately. Results are shown in the graphs as MFI of perforin^low^ (e), MFI of perforin^high^ (f), % of perforin^low^ (g) and % of perforin^high^ (h). Results are presented as dot plots connected with lines to compare state before and after stimulation. Statistical significance was evaluated using the Mann–Whitney test in GraphPad Prism software; *n* = 5 (patients P1–P5; data from P6 were obtained later and are not included in this graph but showed similarly reduced values);  ^∗^ *p* < 0.05. MFI, median fluorescence intensity.

**Figure:**
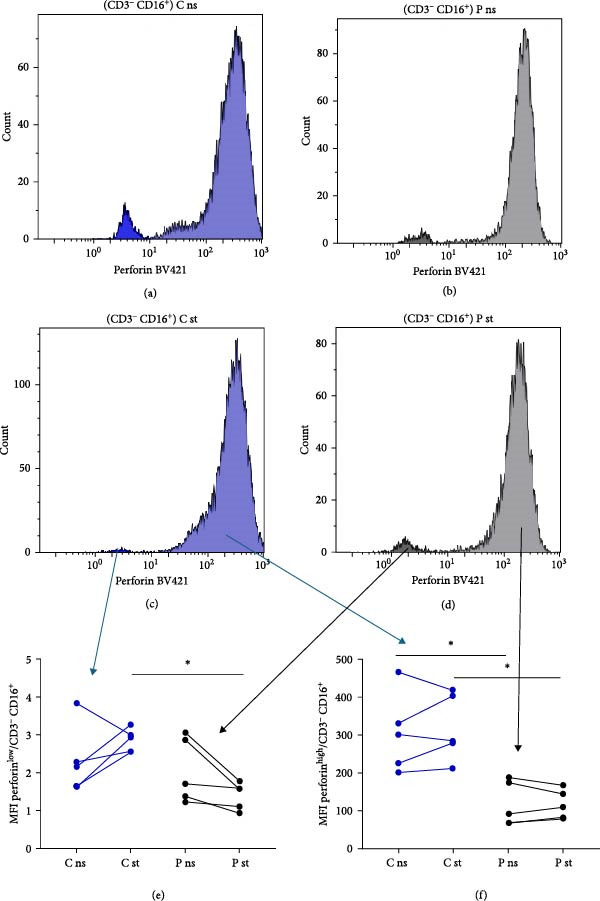

**Figure:**
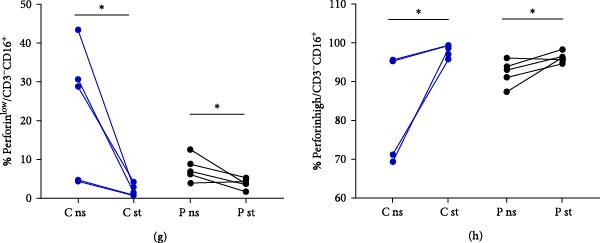

To accurately define these perforin‐expressing subsets, fluorescence minus one (FMO) controls were employed as gating controls. The gating strategy, demonstrated in Supporting information: Figure S1, identified the lower‐expressing peak overlapping with the FMO control by approximately 1.9%; this entire peak was, therefore, considered positive. The chosen gating strategy did not affect the MFI values of high perforin‐expressing populations.

In our analysis of XMEN patients and healthy controls, both subpopulations of CD8^+^ T‐cells were present at comparable frequencies (Figure 2g,h). Upon stimulation, perforin expression significantly increased in the CD8^+^perforin^low^ population of healthy donors; however, no significant increase was observed in the CD8^+^ perforin^low^ population of XMEN patients (Figure 2e). Although the MFI of perforin in the CD8^+^ perforin^high^ population was lower in XMEN patients compared to healthy controls, no significant increase upon stimulation was observed in either group (Figure 2f).

Following stimulation, we observed a decrease in the proportion of NK perforin^low^ cells (Figure 3g) and a corresponding increase in NK perforin^high^ cells (Figure 3h) in both patients and healthy donors, with a comparable magnitude of change between the two groups. However, perforin expression levels within these subsets did not increase upon stimulation in either group. Instead, both NK perforin^low^ and NK perforin^high^ populations in patients exhibited lower perforin expression compared to healthy donors after stimulation (Figure 3e,f). Notably, NK perforin^high^ cells in XMEN patients already showed reduced perforin expression prior to stimulation (Figure 3f). Patient P6 was added to the study cohort at a later stage, and therefore his sample was analysed separately. Although not shown in the Figures 2 and 3, patient P6 also exhibited similarly reduced perforin expression compared to healthy controls, consistent with the findings in other patients.

### 3.5. N‐Glycosylation Analysis

XMEN syndrome is related to defects in N‐glycosylation, thus we decided to investigate if N‐glycosylation is affected also in our patients. We analysed CD8^+^ cells and NK cells from patients P1 and P2, alongside with CD8^+^ cells and NK cells from healthy controls. Flow cytometry analysis confirmed that XMEN disease is associated with impaired N‐glycosylation. Specifically, we identified changes in complex N‐glycans containing a 2,6 branch (detected by PHA‐L binding) and in α2‐6‐sialylated products (detected by SNA binding). In CD8^+^ cells, the binding of PHA‐L and SNA was reduced in patient samples compared to healthy individuals. Similar results were observed for NK cells (Figure 4a–d).

Figure 4Detection of impaired N‐glycosylation. (a, b) Flow cytometric analysis of intracellular N‐glycosylation in CD8^+^ cells. (a) PHA‐L and (b) SNA binding were detected in CD8^+^ cells from the PBMC fraction of patients and healthy volunteers. Cells were gated as LIVE/DEAD^low^, CD45^+^, CD14^−^, CD16^−^, CD3^+^ and CD8^+^. Patient samples were measured in technical duplicates, results are presented in bar graphs as means of MFI with SEM in comparison to four healthy individuals. Representative histograms show the shift between patient samples and a control sample processed on the same day. (c, d) Flow cytometric analysis of intracellular N‐glycosylation in NK cells. (c) PHA‐L and (d) SNA binding were detected in NK cells from the PBMC fraction of patients and healthy volunteers. Cells were gated as LIVE/DEADlow, CD45^+^, CD14^−^, CD16^+^ and CD3^+^. Patient samples were measured in technical duplicates, results are presented in bar graphs as means of MFI with SEM in comparison to four healthy individuals. Representative histograms show the shift between patient samples and a control sample processed on the same day. (e) Detection of N‐glycosylation of transferrin by IEF in serum. Serum samples of patients and healthy volunteers were analysed after saturation of transferrin with Fe (III) on a polyacrylamide gel with a pH gradient of 5–8. The results were visualised by immunofixation using polyclonal rabbit‐anti‐human transferrin antibody and PhastGel Blue R staining. Detected bands were assigned to transferrin glycoforms. Optical density results are shown in the table. Arrows indicate decreased or increased values in patients samples. Transferrin forms: 0–asialotransferrin, 1–monosialotransferrin, 2–disialotransferrin, 3–trisialotransferrin, 4–tetrasialotransferrin, 5–pentasialotransferrin and 6–hexasialotransferrin. SNA, *Sambucus nigra* lectin. CDG, congenital disorder of glycosylation; FITC, fluorescein isothiocyanate; IEF, isoelectric focusing; MFI, median fluorescence intensity; PBMC, peripheral blood mononuclear cell; PHA‐L, *Phaseolus vulgaris* lectin; SEM, standard error of the mean.

**Figure:**
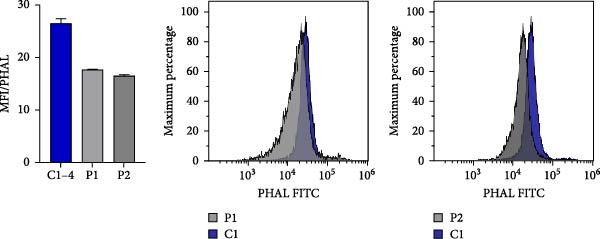
(a)

**Figure:**
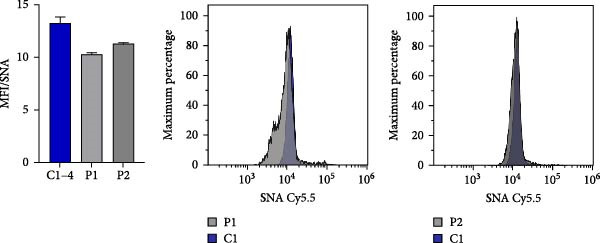
(b)

**Figure:**
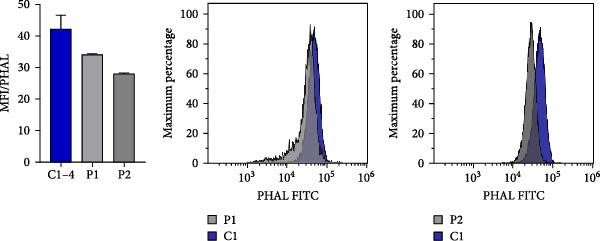
(c)

**Figure:**
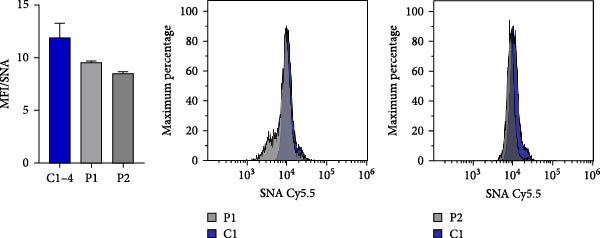
(d)

**Figure:**
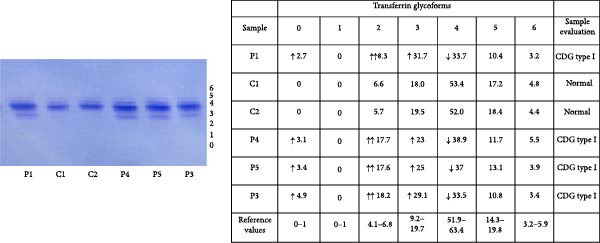
(e)

Isoelectric focusing of serum transferrin in patients P1–P5 showed abnormal glycosylation patterns consistent with congenital disorder of glycosylation. Low‐glycosylated transferrin isoforms are clearly visible on the photo of a polyacrylamide gel in these patients´ samples compared to controls (Figure 4e).

## 4. Discussion

In 2011, Li et al. [1] linked recurrent viral infections, chronic active EBV infection and impaired TCR signalling to a defect in *MAGT1* gene, leading to the establishment of XMEN syndrome. Since then, several patients with a broad spectrum of symptoms have been described [5, 11, 17, 18]. Various studies have reported that *MAGT1* pathogenic variants in XMEN disease result in defective glycosylation, impairing NKG2D expression on NK and T‐cells [5, 19]. Based on these findings, Ravell et al. [8] proposed in 2020 updating XMEN to X‐linked MAGT1 deficiency with increased susceptibility to EBV infection and N‐linked glycosylation defect.

In our study, we present the group of six patients with XMEN who exhibit heterogeneous clinical manifestations yet display typical laboratory signs for the disease. These include decreased surface expression of NKG2D and CD28 on CD8^+^ T‐cells and NK cells, along with an elevated percentage and absolute number of B‐cells, consistent with findings that have been previously published [5, 17].

Two novel variants were identified in patients: a variant c.444dup in exon 3 of the *MAGT1* was present in patients P1 and P2, whereas a variant c.998‐20_1008del at the intron 8–exon 9 junction was identified in Patient P6. Moreover, Patients P3–P5 carried the most common pathogenic variant in *MAGT1*, c.409C >T. The frameshift variant c.444dup and the nonsense c.409C >T both lead to disruptions in protein synthesis at similar positions in exon 3, suggesting potential similar impacts on the clinical phenotype.

Interestingly, despite carrying the same *MAGT1* variant (c.444dup), patients P1 and P2 presented with markedly different clinical phenotypes. Patient P1 predominantly presented with autoimmune cytopenias as the sole and life‐threatening manifestation of the disease. While cytopenias have been documented in several cases of XMEN syndrome, they were not as pronounced and dominating as in the case of P1. Without genetic testing, the diagnosis of XMEN syndrome would have barely been considered in this patient. In contrast, P2 remained largely asymptomatic, displaying only mild viral skin infections. However, their laboratory findings were remarkably similar. Both patients showed reduced surface expression of NKG2D and CD28 on CD8^+^ T‐cells and NK cells, decreased perforin expression, and defective N‐glycosylation. EBV seropositivity was confirmed in both individuals, with no evidence of active infection. Notably, across the entire cohort, all six patients displayed the similarity in laboratory phenotypes—a consistent reduction in CD28 expression on CD8^+^ T‐cells, decreased perforin expression and defective N‐glycosylation in both CD8^+^ T‐cells and NK cells, regardless of clinical severity and various clinical manifestation. These observations highlight the complex interplay between genetic defects and other genetic and environmental factors in shaping disease presentation and suggest that, in XMEN syndrome, functional laboratory parameters may not directly correlate with clinical symptoms. Even in individuals without overt clinical manifestations, characteristic laboratory abnormalities are present, reinforcing the value of immunophenotypic testing in at‐risk individuals. It is noteworthy that, in contrast to patients P3–P6, patient P1 predominantly displayed autoimmune cytopenias as the sole and life‐threatening manifestation of the disease. Hence, akin to other IEI, XMEN syndrome also demonstrates varying degrees of severity in its penetrance.

The diagnostic hallmark of XMEN ‐ decreased NKG2D expression—was observed in all our patients. We detected defects in N‐glycosylation of transferrin, consistent with the N‐linked glycosylation defect described in XMEN syndrome. Decreased perforin expression in XMEN syndrome was recently reported by Wang et al. [13], who described impaired cytotoxic function in both CD8^+^ T‐cells and NK cells as part of the disease pathophysiology. Our findings are in agreement with their report, as we observed consistently reduced perforin expression across all six patients in our cohort, regardless of clinical severity. This reduction was present even in individuals with mild or no clinical symptoms, supporting the notion that cytotoxic dysfunction is aconsistent immunopathological feature of XMEN syndrome.

Previous studies have shown that cytotoxic activity in cytotoxic T‐cells positively correlates with high perforin expression in vivo [20]. Although we were unable to directly assess cytotoxic function in our study, the observed differences in perforin expression—particularly the impaired regulation in CD8^+^ perforin^low^ and NK perforin^low^ populations, as well as the reduced expression in CD8^+^ perforin^high^ and NK perforin^high^ populations in XMEN patients—suggest a likely reduction in cytolytic capacity. This assumption is supported by the findings of Wang et al. [13], who reported reduced cytotoxicity in lymphocytes from XMEN patients, consistent with the observed defects in perforin expression. In addition, partial perforin deficiency has been implicated in an increased susceptibility to haematological malignancies, including leukaemia and lymphoma. A reduced ability to eliminate transformed or virally infected cells may contribute to defective immune surveillance, which could underlie the increased risk of malignancy in these patients [21]. The deficiency in cytotoxic activity may contribute to the susceptibility to recurrent infections and autoimmune complications observed in affected individuals. N‐glycosylation of perforin does not impact its function but is important in perforin transport from Golgi compartment to secretory granules and/or lysosomes [22]. Defect in expression observed by us may be caused by higher degradation of activated non‐glycosylated form of perforin or by degradation due to incorrect position (dysfunction of transport to secretory granules). It is our contention that further investigation is warranted to elucidate the intricacies of this mechanism.

Interestingly, familial hemophagocytic lymphohistiocytosis (FHL), caused by perforin deficiency, shares clinical and pathophysiological similarities with XMEN. In both conditions, CD8^+^ T‐cells and NK cells exhibit significant functional impairment, leading to overlapping clinical features such as susceptibility to viral infections, cytopenias and increased risk of lymphomas and other malignancies. Our results suggest that a defect in perforin expression contributes to the pathogenic mechanism of XMEN, mirroring the immune dysfunction seen in FHL [23].

## 5. Conclusion

This study documents two previously unreported pathogenic variants in the *MAGT1* gene associated with XMEN syndrome. Additionally, an atypical presentation characterised by profound autoimmune cytopenias as the sole clinical manifestation of XMEN in a patient is delineated. Finally, we reaffirmed a deficiency in perforin expression as a contributing factor in the pathogenic mechanism of XMEN syndrome.

## Disclosure

The corresponding authors, on behalf of all contributing authors, hereby declare that the information provided in this disclosure is true and complete to the best of their knowledge and belief.

## Conflicts of Interest

The authors declare no conflicts of interest.

## Author Contributions

The first two authors have contributed equally to this article, and both should be considered first author. The last two authors have contributed equally to this article, and both should be considered corresponding senior author.

## Funding

The study was supported by the Grant NU23‐07‐00170 issued by the Czech Health Research Council and Ministry of Health, Czech Republic. Additionally, it was supported by the institutional support from the Masaryk University, Czech Republic (Grant MUNI/A/1566/2023) and from the University Hospital in Motol, Czech Republic (Grant #00064203).

## Supporting Information

Additional supporting information can be found online in the Supporting Information section.

## Supporting information


**Supporting Informaton** Figure S1. Overlay of FMO perforin control and fully stained sample demonstrating the gating strategy. The lower‐expressing peak overlapped with the FMO control peak by approximately 1.9% and was therefore considered positive. Values in the graphs indicate MFI for each peak. This gating strategy did not affect the MFI values of populations with high perforin expression, highlighting significant differences between healthy donors and patients. (a) Gating of the CD3^+^CD8^+^ population. (b) Gating of the CD3^–^CD16^+^ population.

## Data Availability

The data that support the findings of this study are available from the corresponding authors, Tomas Freiberger and Milos Jesenak, upon reasonable request.
