# Supplementary material for: Atypical Phenotype of Predominant Autoimmune Cytopenia and Impaired Perforin Expression in XMEN Syndrome
Source: J Immunol Res. 2025 Oct 24;2025:3161910. doi: 10.1155/jimr/3161910 (PMC12551146; doi:10.1155/jimr/3161910)
Supplement: Supplementary file 1 — Supporting Informaton Figure S1. Overlay of FMO perforin control and fully stained sample demonstrating the gating strategy. The lower‐expressing peak overlapped with the FMO control peak by approximately 1.9% and was therefore considered positive. Values in the graphs indicate MFI for each peak. This gating strategy did not affect the MFI values of populations with high perforin expression, highlighting significant differences between healthy donors and patients. (a) Gating of the CD3+CD8+ population. (b) Gating of the CD3–CD16+ population. [file JIMR-2025-3161910-s001.zip › sup. figure 1.pptx]

## Slide 1
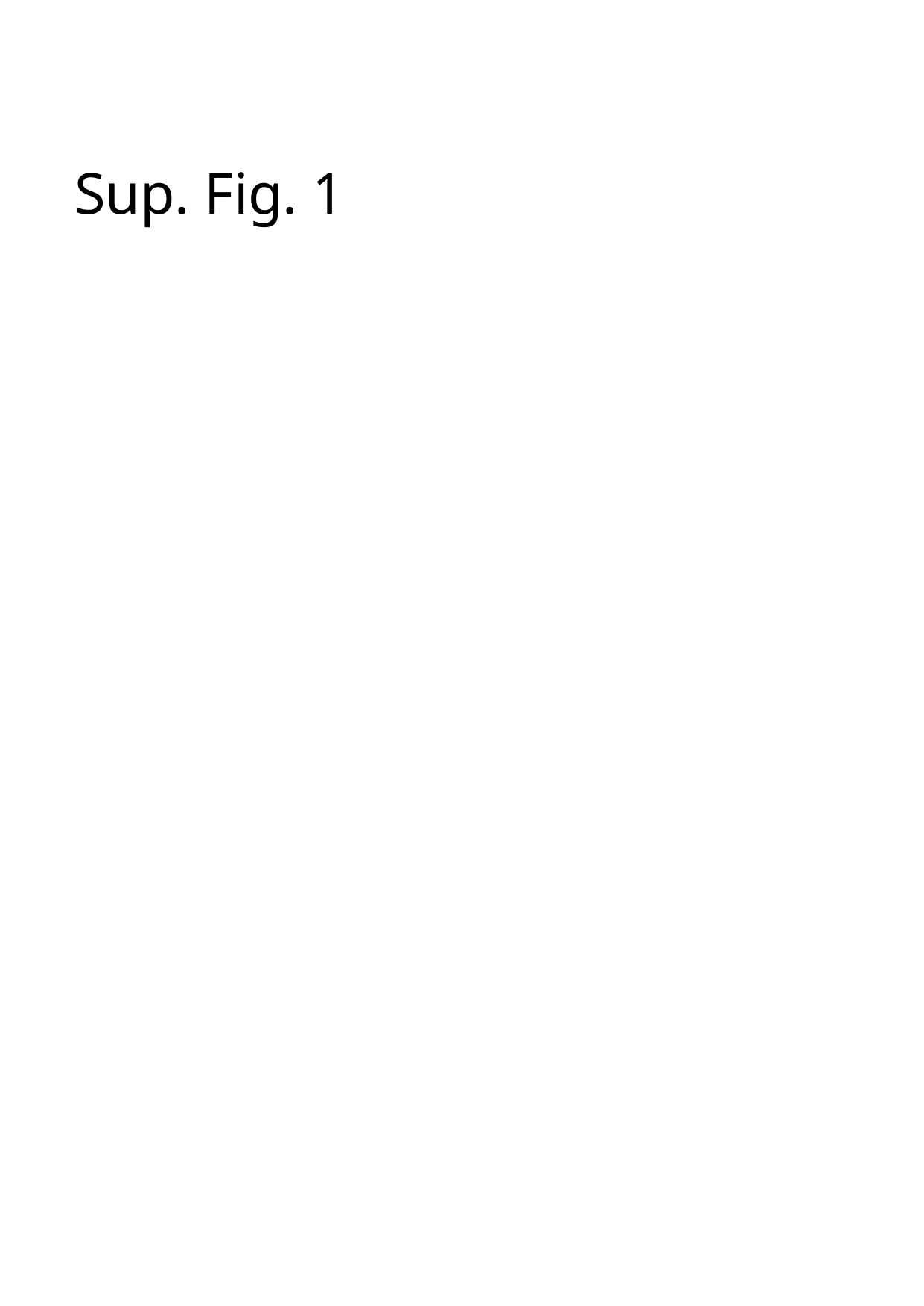

# Sup. Fig. 1

## Slide 2
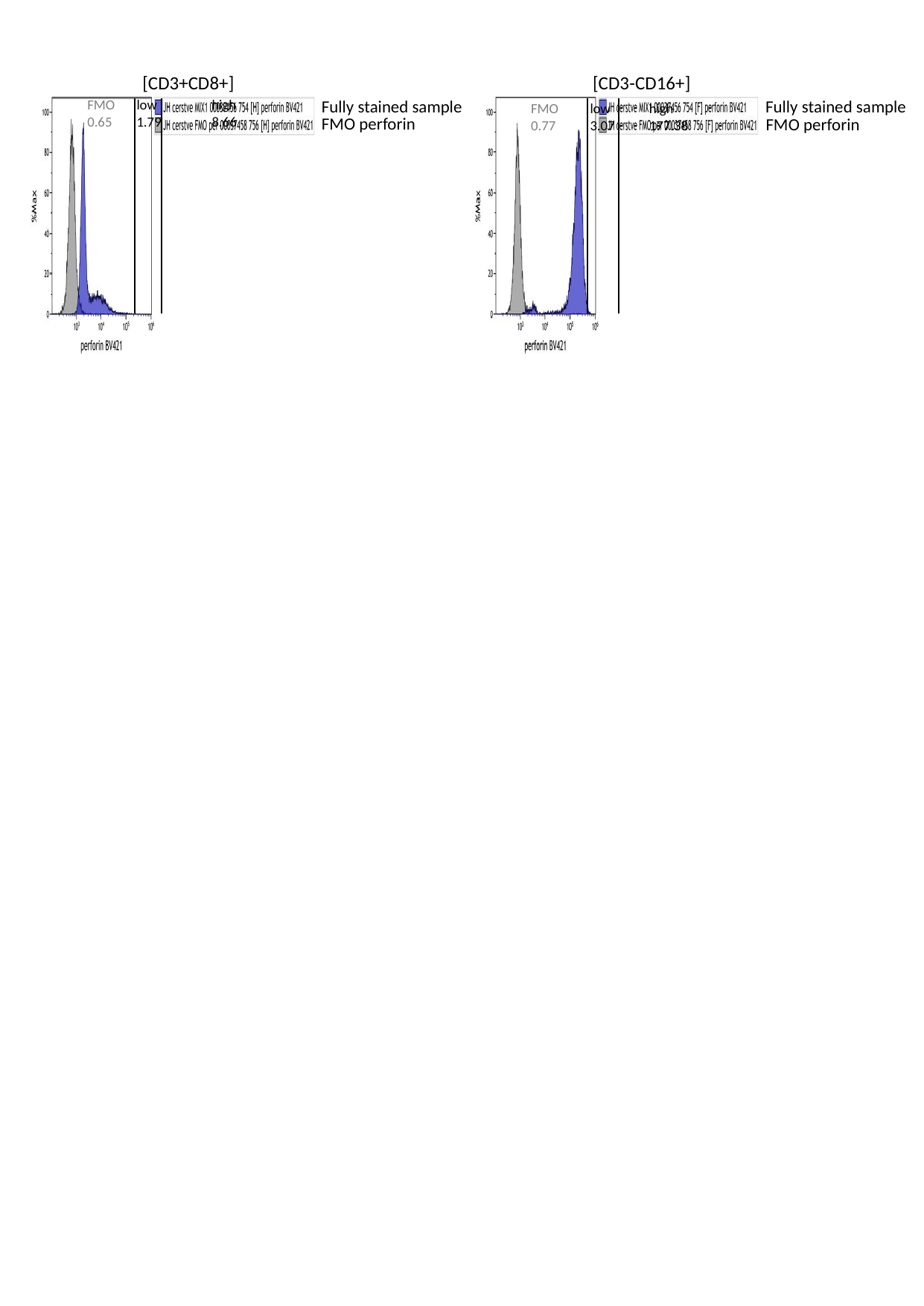

[CD3-CD16+]
[CD3+CD8+]
FMO
0.65
low
1.79
high
8.66
Fully stained sample
Fully stained sample
FMO
0.77
low
3.07
high
177.38
FMO perforin
FMO perforin
