# Supplementary material for: Atypical Phenotype of Predominant Autoimmune Cytopenia and Impaired Perforin Expression in XMEN Syndrome
Source: J Immunol Res. 2025 Oct 24;2025:3161910. doi: 10.1155/jimr/3161910 (PMC12551146; doi:10.1155/jimr/3161910)
Supplement: Supplementary file 1 — Supporting Informaton Figure S1. Overlay of FMO perforin control and fully stained sample demonstrating the gating strategy. The lower‐expressing peak overlapped with the FMO control peak by approximately 1.9% and was therefore considered positive. Values in the graphs indicate MFI for each peak. This gating strategy did not affect the MFI values of populations with high perforin expression, highlighting significant differences between healthy donors and patients. (a) Gating of the CD3+CD8+ population. (b) Gating of the CD3–CD16+ population. [file JIMR-2025-3161910-s001.zip › Supporting_information.docx]

**Supporting information**

**NGS Analysis**

Various NGS procedures specified below were used to detect causal defect in patient P1, P3, and P6.

**Patient P1 NGS analysis**

Library was prepared using SureSelect Custom Panel and SureSelect XT HS2 DNA Reagent Kit (Agilent). Custom Panel was targeted on IEI genes (see below). The prepared samples were sequenced using NextSeq Illumina.

Library preparation was performed using the SureSelect Custom Panel (Agilent) and compatible reagents. The custom panel was specifically designed to target genes associated with inborn errors of immunity (IEI), as listed below. Prepared libraries were sequenced on the Illumina NextSeq platform.

**Patient P1 IEI targeted gene list**

AK2, ABCB1, ACD, ACOD1, ACP5, ACTB, ADA, ADA2, ADAR, ADAM17, AICDA, AIRE, ALPI, ANKRD26, ANKZF1, AP3B1, APOL1, AP1S3, ARHGEF1, ARPC1B, ATG16L1, ATP6AP1, B2M, BACH2, BCL10, BCL11B, BLM, BLNK, BLOC1S6, BRCA2, BRIP1, BTK, C1QA, C1QB, C1QC, C1R, C1S, C2, C3, C5, C6, C7, C8A, C8B, C8G, C8orf34, C9, CARD11, CARD14, CARD16, CARD9, CARMIL2, CASP10, CASP8, CCBE1, CD19, CD247, CD27, CD28, CD3D, CD3E, CD3G, CD40, CD40LG, CD46, CD55, CD59, CD70, CD79A, CD79B, CD81, CD8A, CDCA7, CEBPE, CFB, CFD, CFH, CFHR1, CFHR2, CFHR3, CFHR4, CFHR5, CFI, CFP, CIB1, CIITA, CLCN7, CLPB, COL7A1, COPA, CORO1A, CR2, CSF2RA, CSF2RB, CSF3R, CTC1, CTLA4, CTPS1, CTSC, CXCR4, CYBA, CYBB, CYBC1, CYCS, DCLRE1B, DCLRE1C, DEF6, DKC1, DNAJC21, DNASE1L3, DNMT3B, DOCK2, DOCK8, DTNBP1, DUOX2, ELANE, ERBIN, ERCC4, ERCC6L2, ETV6, EXTL3, F12, FAAP24, FADD, FANCA, FANCB, FANCC, FANCD2, FANCE, FANCF, FANCI, FANCL, FANCM, FAS, FASLG, FCGR3A, FCGR3B, FCHO1, FCN3, FERMT1, FERMT3, FNIP1, FOXN1, FOXP3, FPR1, FYB1, G6PC3, G6PD, GATA2, GFI1, GINS1, GUCY2C, HAVCR2, HAX1, HELLS, HMOX1, HPS1, HPS3, HPS4, HPS5, HPS6, HSPA1L, HYOU1, ICOS, ICOSLG, IFIH1, IFNAR1, IFNAR2, IFNGR1, IFNGR2, IGHM, IGKC, IGLL1, IKBKB, IKBKG, IKZF1, IL1RN, IL10, IL10RA, IL10RB, IL12B, IL12RB1, IL17A, IL17F, IL17RA, IL17RC, IL18BP, IL21, IL21R, IL22, IL23R, IL2RA, IL2RB, IL2RG, IL36RN, IL6, IL6R, IL6ST, IL7, IL7R, INAVA, INO80, IRAK1, IRAK4, IRF2BP2, IRF3, IRF4, IRF5, IRF7, IRF8, IRF9, IRGM, ISG15, ITCH, ITGAM, ITGB2, ITK, JAGN1, JAK1, JAK3, KMT2A, LAMTOR2, LAT, LCK, LIG1, LIG4, LPIN2, LRBA, LYST, MAD2L2, MAGT1, MALT1, MAP3K14, MASP2, MCM4, MEFV, MFAP5, MOGS, MRTFA, MS4A1, MSH6, MSN, MTHFD1, MVK, MYD88, MYH9, MYSM1, NBN, NCF1, NCF2, NCF4, NCSTN, NFAT5, NFE2L2, NFKB1, NFKB2, NFKBIA, NFKBIB, NHEJ1, NHP2, NLRC4, NLRP1, NLRP3, NLRP12, NOD2, NOP10, NOX1, NPC1, NSMCE3, OAS1, ORAI1, OSTM1, OTULIN, PALB2, PARN, PEPD, PGM3, PIK3CD, PIK3CG, PIK3R1, PLA2G4A, PLCG2, PLEKHM1, PLG, PMS2, PNP, POLA1, POLD1, POLD2, POLE, POLE2, POLR3A, POLR3C, POLR3F, POMP, PRDM1, PRF1, PRKCD, PRKDC, PSEN1, PSENEN, PSMA3, PSMB4, PSMB8, PSMB9, PSMG2, PSTPIP1, PTCRA, PTEN, PTPRC, RAB27A, RAC2, RAD51, RAD51C, RAG1, RAG2, RANBP2, RASGRP1, RBCK1, REL, RELA, RELB, RFWD3, RFX5, RFXANK, RFXAP, RHOH, RIPK1, RMRP, RNASEH2A, RNASEH2B, RNASEH2C, RNF168, RNF186, RNF31, RNU4ATAC, RORC, RPSA, RTEL1, SAMD9, SAMD9L, SAMHD1, SART3, SBDS, SEC61A1, SEMA3E, SERPING1, SH2D1A, SH3BP2, SH3KBP1, SHARPIN , SKIV2L, SLC29A3, SLC35C1, SLC37A4, SLC39A7, SLC46A1, SLC7A7, SLC9A3, SLCO2A1, SLX4, SMARCAL1, SMARCD2, SMURF1, SNM1, SNX10, SOCS4, SP110, SPINK5, SPPL2A , SRC, SRP54, SRP72, STAT1, STAT2, STAT3, STAT5B, STIM1, STK4, STN1, STX11, STXBP2, STXBP3, TAP1, TAP2, TAPBP, TAZ, TBK1, TBX1, TCF3, TCIRG1, TCN2, TERC, TERT, TFRC, TGFB1, TGFBR1, TGFBR2, THBD, TICAM1, TINF2, TIRAP, TLR1, TLR10, TLR2, TLR3, TLR4, TLR5, TLR6, TLR7, TLR8, TLR9, TMC6, TMC8, TMEM173, TNFAIP3, TNFRSF11A, TNFRSF13B, TNFRSF13C, TNFRSF1A, TNFRSF4, TNFRSF9, TNFSF11, TNFSF12, TNFSF13, TNFSF15, TOP2B, TP53, TPP1, TPP2, TRAC, TRAF3, TRAF3IP2, TREX1, TRIM22, TRNT1, TTC37, TTC7A, TYK2, UBE2T, UNC119, UNC13D, UNC93B1, UNG, USB1, USP18, VPS13B, VPS45, WAS, WASF2, WDR1, WIPF1, WRAP53, XIAP, XRCC2, XRCC9, ZAP70, ZBTB24, ZNF341

**Patient P3 NGS analysis**

Library preparation was performed using SureSelect Custom Panel and SureSelect XT HS2 DNA Reagent Kit (Agilent). The custom panel was specifically designed to target genes associated with inborn errors of immunity (IEI), as listed below. Prepared libraries were sequenced on the Illumina 550 system.

**Patient P3 IEI targeted gene list**

ACD, ACP5, ACTB, ADA, ADA2, ADAM17, ADAR, AICDA, AIRE, AK2, ALPI, AP1S1, AP1S3, AP3B1, AP3D1, APOL1, ARHGEF1, ARPC1B, ATM, ATP6AP1, B2M, BACH2, BCL10, BCL11B, BLM, BLNK, BRCA1, BRCA2, BRIP1, BTK, C1QA, C1QB, C1QC, C1R, C1S, C2, C3, C4A, C4B, C5, C6, C7, C8A, C8B, C8G, C9, CARD11, CARD14, CARD9, CARMIL2, CASP10, CASP8, CCBE1, CCDC22, CD19, CD247, CD27, CD3D, CD3E, CD3G, CD40, CD40LG, CD46, CD48, CD55, CD59, CD70, CD79A, CD79B, CD81, CD8A, CDC42, CDCA7, CEBPE, CFB, CFD, CFH, CFHR1, CFHR2, CFHR3, CFHR4, CFHR5, CFI, CFP, CFTR, CHD7, CIB1, CIITA, CLCN7, CLPB, COL7A1, COPA, CORO1A, CR2, CSF2RA, CSF2RB, CSF3R, CTC1, CTLA4, CTPS1, CTSC, CXCR4, CYBA, CYBB, CYBC1, DBR1, DCLRE1C, DEF6, DKC1, DNAJC21, DNASE1L3, DNASE2, DNMT3B, DOCK2, DOCK8, DUOX2, EFL1, ELANE, EOMES, EPG5, ERBIN, ERCC4, ERCC6L2, EXTL3, FAAP24, FADD, FANCA, FANCB, FANCC, FANCD2, FANCE, FANCF, FANCG, FANCI, FANCL, FANCM, FAS, FASLG, FAT4, FCGR3A, FCHO1, FCN3, FERMT1, FERMT3, FOXN1, FOXP3, FPR1, G6PC3, G6PD, GATA2, GFI1, GINS1, HAVCR2, HAX1, HCK, HELLS, HMOX1, HYOU1, ICOS, ICOSLG, IFIH1, IFNAR1, IFNAR2, IFNG, IFNGR1, IFNGR2, IGHM, IGKC, IGLL1, IKBKB, IKBKG, IKZF1, IKZF2, IL10, IL10RA, IL10RB, IL12B, IL12RB1, IL12RB2, IL17F, IL17RA, IL17RC, IL18BP, IL1RN, IL21, IL21R, IL23R, IL2RA, IL2RB, IL2RG, IL36RN, IL6R, IL6ST, IL7R, INO80, IRAK1, IRAK4, IRF2BP2, IRF3, IRF4, IRF7, IRF8, IRF9, ISG15, ITCH, ITGB2, ITK, JAGN1, JAK1, JAK3, KDM6A, KMT2A, KMT2D, LAMTOR2, LAT, LCK, LIG1, LIG4, LPIN2, LRBA, LYN, LYST, MAD2L2, MAGT1, MALT1, MAP3K14, MASP2, MCM4, MEFV, MOGS, MRTFA, MS4A1, MSH6, MSN, MTHFD1, MVK, MYD88, MYSM1, NBAS, NBN, NCF1, NCF2, NCF4, NCKAP1L, NCSTN, NFAT5, NFE2L2, NFKB1, NFKB2, NFKBIA, NHEJ1, NHP2, NLRC4, NLRP1, NLRP12, NLRP3, NOD2, NOP10, SNORA31, NSMCE3, OAS1, ORAI1, OSTM1, OTULIN, PALB2, PARN, PEPD, PGM3, PIK3CD, PIK3CG, PIK3R1, PLCG2, PLEKHM1, PMS2, PNP, POLA1, POLD1, POLD2, POLE, POLE2, POLR3A, POLR3C, POLR3F, PRF1, PRKCD, PRKDC, PSEN1, PSENEN, PSMB8, PSMG2, PSTPIP1, PTEN, PTPRC, RAB27A, RAC2, RAD51, RAD51C, RAG1, RAG2, RANBP2, RASGRP1, RBCK1, REL, RELA, RELB, RFWD3, RFX5, RFXANK, RFXAP, RHOH, RIPK1, RMRP, RNASEH2A, RNASEH2B, RNASEH2C, RNF168, RNF31, RNU4ATAC, RORC, RPSA, RTEL1, SAMD9, SAMD9L, SAMHD1, SBDS, SEC61A1, SEMA3E, SERPING1, SH2D1A, SH3BP2, SH3KBP1, SKIV2L, SLC29A3, SLC35C1, SLC37A4, SLC39A7, SLC46A1, SLC7A7, SLX4, SMARCAL1, SMARCD2, SNX10, SP110, SPINK5, SPPL2A, VIII.54, VIII.72, STAT1, STAT2, STAT3, STAT5B, STIM1, STING1, STK4, STN1, STX11, STXBP2, TAP1, TAP2, TAPBP, TAZ, TBK1, TBX1, TCF3, TCIRG1, TCN2, TERC, TERT, TFRC, TGFB1, TGFBR1, TGFBR2, THBD, TICAM1, TINF2, TIRAP, TLR1, TLR10, TLR2, TLR3, TLR4, TLR5, TLR6, TLR7, TLR8, TLR9, TMC6, TMC8, TNFAIP3, TNFRSF11A, TNFRSF13B, TNFRSF13C, TNFRSF1A, TNFRSF4, TNFRSF9, TNFSF11, TNFSF12, TOM1, TOP2B, TP53, TPP2, TRAC, TRAF3, TRAF3IP2, TREX1, TRIM22, TRNT1, TTC37, TTC7A, TYK2, UBE2T, UNC13D, UNC93B1, UNG, USB1, USP18, VPS13B, VPS45, WAS, WDR1, WIPF1, WRAP53, XIAP, XRCC2, ZAP70, ZBTB24, ZNF341.

**Patient P6 NGS analysis**

Libraries were prepared using Illumina DNA Prep with Enrichment (Illumina) and Twist Alliance VCGS Exome (Twist). The prepared samples were sequenced using NextSeq Illumina.

Library preparation was performed using the SureSelect Custom Panel (Agilent) and compatible reagents. The custom panel was specifically designed to target genes associated with inborn errors of immunity (IEI), as listed below. Prepared libraries were sequenced on the Illumina NextSeq 550 system.

**Patient P6 NGS analysis**

Library preparation was performed using Twist Alliance VCGS Exome (Twist) and Illumina DNA Prep with Enrichment (Illumina). Prepared libraries were sequenced on the NovaSeq 600 (Illumina).

**Fig. S1**: **Overlay of FMO perforin control and fully stained sample demonstrating the gating strategy**. The lower-expressing peak overlapped with the FMO control peak by approximately 1.9% and was therefore considered positive. Values in the graphs indicate MFI for each peak. This gating strategy did not affect the MFI values of populations with high perforin expression, highlighting significant differences between healthy donors and patients.
(a) Gating of the CD3+CD8+ population; (b) Gating of the CD3–CD16+ population.
